# Extracting Knowledge of 2D‐Hybrid Halide Perovskite Materials: A Data Mining Approach

**DOI:** 10.1002/open.202500606

**Published:** 2026-03-12

**Authors:** Guadalupe Castro, Miriam Pescador‐Rojas, Joel Ireta

**Affiliations:** ^1^ Departamento de Química Universidad Autónoma Metropolitana‐Iztapalapa Ciudad de México Mexico; ^2^ Escuela Superior de Cómputo Instituto Politécnico Nacional Ciudad de México Mexico

**Keywords:** bandgap, data mining, perovskite, semiconductors

## Abstract

2D‐hybrid halide perovskites are semiconductor materials with excellent optical properties that have been widely studied due to their potential as materials applicable to green energy production. Data mining techniques are powerful tools to recognize and extract relevant patterns from databases. In this study, data mining techniques are used to extract relationships among geometric characteristics, composition, and the bandgap of 2D‐hybrid halide perovskites. Our analysis reveals patterns that connect the chemical properties of the organic spacer cation with the bandgap, like the distance between the halogen in the perovskite and the terminal nitrogen of the interlayer organic cation, and the relation among the type of organic interlayer cation, the interlayer distance, and the perovskite layer phase. Furthermore, it is found that aromatic cations lead to bandgaps between 2.2 and 2.4 eV. These results are consistent with previous experimental reports and provide insight into the structure–property relationships, thus illustrating the utility of data mining techniques for extracting valuable knowledge to optimize and design new materials with improved properties.

## Graphical abstract

Data mining is used to uncover patterns among the composition, structure, and physicochemical properties of 2D‐hybrid halide perovskites. The results show the significance of the chemical nature of spacer cations, which affects the structural parameters and the bandgap. These relationships contribute to the understanding of these semiconductors, which may be essential for optimizing and designing new materials relevant to the energy sector.

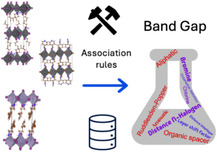

## 1 Introduction

**FIGURE 1:**
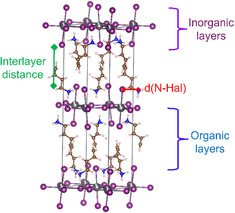
Unit cell of 2D‐hybrid halide perovskites. Color code: halogen atoms (X) in purple, metallic center atoms (M) in gray, carbon atoms in brown, nitrogen atoms in blue, and hydrogen atoms in white. The red line denotes the distance between the halogen and nitrogen atoms d(X–H). The green line corresponds to the distance between inorganic layers (InterL).

In recent years, two‐dimensional (2D)‐hybrid halide perovskites have attracted wide attention due to their structural flexibility, stability, and excellent optoelectronic and photovoltaic properties [1, 2, 3]. As illustrated in Figure 1, these materials have a layered structure which provokes unique properties and a broad range of potential applications, such as solar cells, light‐emitting diodes, transistors, and green energy production [3, 4, 5, 6]. The general formula of 2D‐hybrid halide perovskites is (L)_2_(A)_*n*−1_M_*n*_X_3*n* + 1_, where L is an organic spacer cation, A is a small organic cation, M is a metal cation, X is a halide anion, and *n* is an integer [4]. The bandgap is one of the most relevant properties for applying materials in solar cells and electronic devices. For 2D‐hybrid perovskite materials, it has been suggested that the composition and thickness of the inorganic layer influence their bandgap [1, 2, 3, 4, 5, 6]. Hence, one may wonder if the organic cations also have an effect on it.

The organic spacer cations in layered perovskites can be aliphatic or aromatic compounds, with ammonium cations, such as phenylethylammonium (PEA) and butylammonium (BA), being the most used ones [7]. It has been proposed that these organic spacer cations mainly influence the structural properties of 2D‐hybrid perovskites [7, 8] through interactions such as hydrogen bonds, halogen bonds, and van der Waals forces [7, 8, 9, 10, 11].

In recent years, 2D‐hybrid halide perovskites have attracted wide attention due to their structural flexibility, stability, and excellent optoelectronic and photovoltaic properties [1, 2, 3]. As illustrated in Figure 1, these materials have a layered structure which provokes unique properties and a broad range of potential applications, such as solar cells, light‐emitting diodes, transistors, and green energy production [3, 4, 5, 6]. The general formula of 2D‐hybrid halide perovskites is (L)_2_(A)_*n *− 1_M_*n*_X_3*n* + 1_, where L is an organic spacer cation, A is a small organic cation, M is a metal cation, X is a halide anion, and *n* is an integer [4]. The bandgap is one of the most relevant properties for applying materials in solar cells and electronic devices. For 2D‐hybrid perovskite materials, it has been suggested that the composition and thickness of the inorganic layer influence their bandgap [1, 2, 3, 4, 5, 6]. Hence, one may wonder if the organic cations also have effect on it.

The organic spacer cations in layered perovskites can be aliphatic or aromatic compounds, being ammonium cations as PEA and BA the most used ones [7]. It has been proposed that these organic spacer cations mainly influence the structural properties of 2D‐hybrid perovskites [7, 8] through interactions such as hydrogen bonds, halogen bonds, and van der Waals forces [7, 8, 9, 10, 11]. Also, it has been reported that organic cations can modulate the conductivity of hybrid perovskites [7]. Furthermore, the number of cycles of the aromatic cations seems to alter the bandgap and its alignment [9]. Therefore, such organic compounds may also influence the optoelectronic properties of these materials. Yet, our comprehension of the effect of organic cations on the optoelectronic properties of layered perovskites is still limited [9].

**FIGURE 2:**
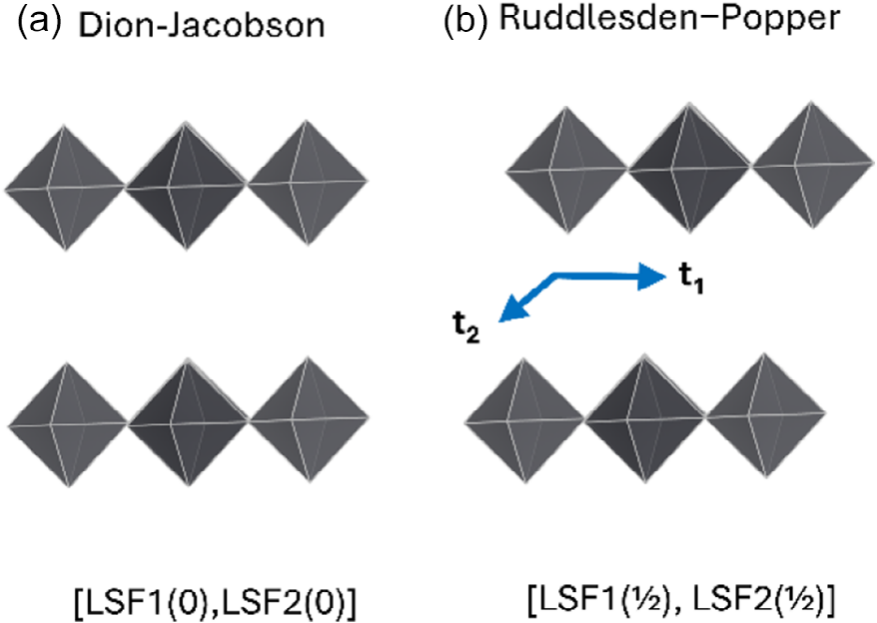
Phases of the 2D‐hybrid halide perovskites described by the layer shift factor (LSF(**t_1_**, **t_2_**)). Vectors **t_1_** and **t_2_** are orthogonal to the normal vectors of the layers and orthogonal between them.

Recently, it has been suggested that the interlayer distance (InterL, see Figure 1), the structural stability, and the optoelectronic properties are related to the phase of these materials (Figure 2) [2, 7, 12], which is connected to the relative position of the inorganic layers. The most studied phases are the Dion−Jacobson (DJ) and the Ruddlesden−Popper (RP). The DJ phase corresponds to eclipsed layers as viewed along the vectors normal to them (Figure 2a), while the RP phase results from a half‐octahedra displacement between the two inorganic layers (Figure 2b) [2, 7, 13]. However, several structures with intermediate shifts between layers are not considered in the mentioned classification. For this reason, it has been proposed a quantitative descriptor of the shift between such layers named lyer shift factor, LSF(**t**_**1**_ **, t**_**2**_), where **t**_**1**_ and **t**_**2**_ are two translational vectors orthogonal between them and to the normal vectors of the inorganic layers. LSF(**t**_**1,**_ **t**_**2**_) takes a maximum value of 0.5, which corresponds to half of the octahedron displacement. To denote these vectors, we refer to them as LSF1 for **t**_**1**_ and LSF2 for **t**_**2**_ [14]. As shown in Figure 2, the phase DJ corresponds to [LSF1(0), LSF2(0)] and RP to [LSF1(1/2), LSF2(1/2)].

Recently, Marchenko et al. published a database of 2D‐hybrid halide perovskites that includes experimental bandgaps, experimental structural parameters, and features related to the composition of these materials [15]. Using a machine learning model, these authors predicted bandgaps for such materials. They also correlated some characteristics of these materials with their experimental bandgaps to identify which ones most significantly influence them. In data mining and machine learning, it is common to refer to a descriptor as an element of the set of material characteristics used in the analysis. Marchenko et al. correlated descriptors of the inorganic layers like the number of layers, the M–X–M angle, and the penetration of organic cations into the inorganic layer with the bandgaps [15]. However, that analysis did not consider the chemical features of the organic spacer cations nor any descriptor corresponding to the phase of these materials [15]. Here, we aim to find correlations among descriptors of the organic spacer cations, the phase of these materials, and their bandgaps by means of data mining procedures.

Data mining and machine learning techniques have been widely employed in materials science to uncover new knowledge by revealing significant hidden patterns that are key to materials design [15, 16, 17, 18, 19, 20]. It uses various methods to extract valuable information from a dataset of the material properties by generating association rules, which are logical expressions of the form Ant → Con, where Ant stands for antecedent and Con for consequent. Ant and Con are constituted by items. Each item is generated by grouping descriptors of a material feature in an interval. The intersection of Ant and Con is an empty set. Thus, Ant and Con are disjoint item sets, meaning that they do not share any item. Association rules can be obtained using the Apriori method [21]. This technique helps to identify correlations among numerical data related to physicochemical properties, structural measurements, and categorical details such as functional groups, elements, and types of phases. Particularly, the Apriori algorithm is adequate to extract relevant information from small datasets [21, 22, 23]. For instance, Can and Yildirim used association rules obtained from a small dataset with the Apriori algorithm to identify the key factors influencing high hydrogen production from perovskites [23].

In this work, we look for key descriptors that affect the bandgap of 2D‐hybrid halide perovskites. Data mining techniques, including association rules, are thus used to uncover patterns among composition, structural characteristics, and physicochemical properties of these perovskites. It is taken into account information related to the chemical characteristics of the organic spacer cations and descriptors representing the perovskite phases. In this way, the influence of such structural properties on the bandgap of 2D‐hybrid halide perovskites is revealed. The association rules found here highlight the significance of the interaction between organic compounds and inorganic layers. Additionally, the results link the composition of these materials with their phase and other structural parameters, which may be crucial for the functionality of these semiconductors in energy generation and for the development of electronic devices.

## 2 Materials and Methods

**FIGURE 3:**
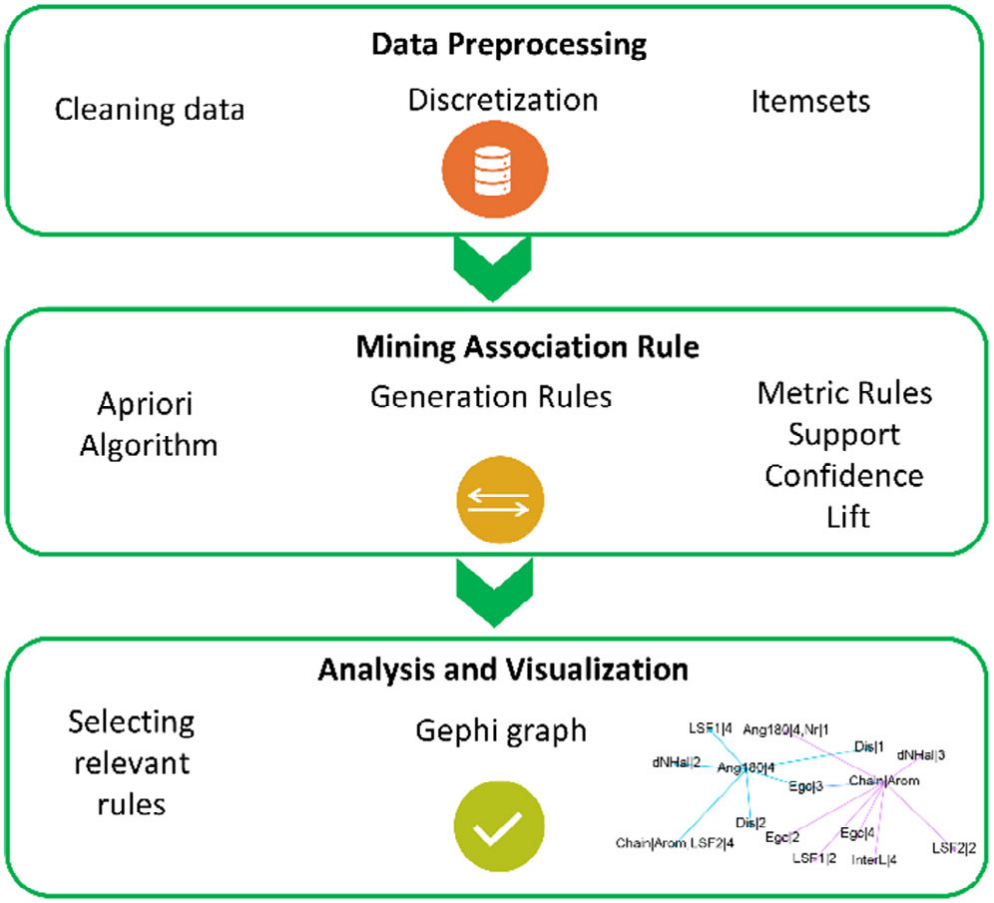
Description of the methodology applied to obtain the analyzed association rules.

The methodology employed comprises three stages (Figure 3): i) data preprocessing to generate items, ii) generation of association rules using the apriori method, and iii) analysis and visualization of the relevant rules. The initial preprocessing step involves selecting a dataset of characteristics that encompasses compositional data, structural information, and experimental (*E*_g_) and theoretical (*E*_gc_) bandgaps [15]. The selected dataset contains 171 materials and 28 descriptors (Table S1). As shown in Table 1, the *E*_gc_ values correspond to the predicted ones using a machine learning regression, as reported by Marchenko et al. [15] We notice that the range of variation of the *E*_gc_ values is smaller than that of the *E*_g_ ones. The former varies between 1.79 and 3.52 eV and the latter from 1.57 to 5.29 eV, i.e., *E*_gc_ values obtained by Marchenko et al. tend to subestimate the *E*_g_ ones, as it is shown by the dispersion plot reported in Figure S1.

**TABLE 1:** Selected set of descriptors considered in the database.

Descriptor	Description	Interval
LSF1	Layer shift factor‐translation 1 (shifting octahedral layers with respect to **t**_**1**_)	0.01–0.5
LSF2	Layer shift factor‐translation 2 (shifting perpendicular to **t**_**1**_)	0.02–0.5
*E*_gc_	Calculated bandgaps energy	1.79–3.52 eV
*E*_g_	Experimental bandgaps energy	1.57–5.29 eV
*A*	*a* unit cell parameter	5.8–74.6 Å
*C*	*c* unit cell parameter	7.8–68.7 Å
Beta	beta unit cell parameter (angle)	69.6°–127.6°
Dis	Octahedral distortion Δd (Alonso et al. [24])	3.21E−8‐0.032
InterL	Distance interlayer	2.6–30.98 Å
Nlay	Layer number	0–7
dNHal	Mean distance between N terminal and halogen	2.96–4.54 Å
Ang180	Mean of angles of octahedral near to 180 (axial)	148.97°–180°
Phase	RP or DJ phase	DJ, RP, else
Hal	Halogen	I, Br, Cl
Cinter	Cation intralayer	None, methylammonium, formamidinium, Cs, ethylammonium
Sym	Symmetry	36 space groups

The full list of descriptors are given in Table S1, Supporting Information. LSF1, LSF2, and Dis are dimensionless predictors.

**FIGURE 4:**
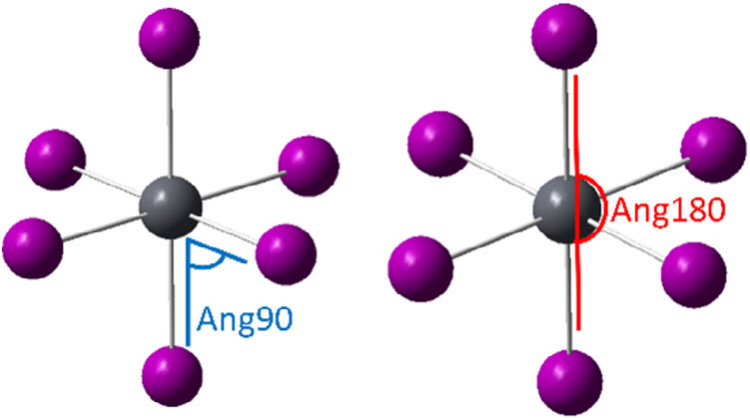
Angles axials (Ang180) and equatorials (Ang90) of the octahedral structure of perovskites.

In addition to the bandgap, other descriptors considered are the classification of phases RP and DJ, LSF1 and LSF2 (see Table 2), the number of inorganic layers (Nlay), InterL, and the average distance between the nitrogen atom of the organic spacer cation and the adjacent halogen atom of the inorganic layer (dNHal). The distortion (Dis) of the octahedral structure around the metallic centers is also included. Dis in perovskites is a geometric characteristic representative of the inorganic layer. As shown in Figure 4, the octahedra contain a center M, which generally is Pb, and six halogens, one at each of its corners. Dis is evaluated by the following equation:

**TABLE 2:** Predictors related to the organic spacer cations.

Predictor	Description	Domain
Chain	Chain organic type	Aliphatic, aromatic
NCchain	Number of C in chain organic	0–12
NN	Number of N terminals in chain organic	0–5
Nr	Number of rings in the organic compound	0–5
NCr	Number of C in ring in the organic compound	0–6
Qc	Formal charge of organic cation	1, 2

(1) $$\mathrm{Dis}=\frac{1}{6}\sum {\left[\frac{d_{n}-d}{d}\right]}^{2}$$

where $d_{n}$ represents each M–X distance and d is the mean of the six $d_{n}$ values. The latter was proposed by Alonso et al. and is frequently used in the study of layered perovskites [24]. The angles inside the octahedral (Figure 4) are also considered to describe the distortion of the octahedral structure. Ang90 corresponds to the mean of angles (equatorials) around 90°, and Ang180 corresponds to the mean of angles around 180° (axials). Hence, the octahedral structure of inorganic layers is accounted for with three geometrical descriptors and features of its composition, such as the metallic center (M) and the halogen (Hal), with lead and iodine being the most common elements, respectively.

The 2D‐hybrid halide perovskites in the dataset contain 90 different organic spacer cations. Despite the overwhelming variety of organic cations used, taking them into account may reveal important relationships with the structural and electronic properties of these perovskites [10].

Table 2 shows the descriptors proposed to capture the chemical features of each organic cation. These descriptors classify them based on the type of carbon chain (Chain), which can be either aliphatic or aromatic, the number of carbon atoms in the chain (NCchain), the number of carbon rings (Nring), and the number of carbon atoms in those rings (NCr). The chain is crucial, as it has been reported that the type of chain can influence the optoelectronic properties and stability of these materials [7]. The size of the organic compounds is indirectly assessed with NCchain and Nring. Here, we have found that these descriptors together with NCr facilitate the identification of patterns related to the chemical nature of the organic spacer cations and serve to link them to structural and physicochemical properties of the perovskites, such as the bandgap.

The numeric data associated with the descriptors are normalized, i.e., values range from zero to one, and discretized using quintiles to create balanced groups with similar frequency values for each descriptor (Table S2). Partitioning a variable into five intervals with approximately equal numbers of observations ensures a balanced distribution across bins, thereby mitigating issues associated with data skewness and sparsity in specific ranges. This balanced allocation enhances the robustness of statistical analyses and diminishes the impact of outliers, as extreme values are absorbed within broader categories rather than disproportionately influencing the variability of continuous measures. For instance, *E*_g_ is divided into five groups (Table 3), resulting in five items labeled with the predictor “*E*_g_” and the corresponding quintile number.

**TABLE 3:** Items generated through the discretization of the *E*_g_ descriptor.

Item|bin	Interval, eV	Frequency
*E*_g_|1	1.57–1.93	29
*E*_g_|2	1.96–2.14	38
*E*_g_|3	2.18–2.38	35
*E*_g_|4	2.39–2.77	34
*E*_g_|5	2.79–5.29	35

The Apriori algorithm employs the items obtained through preprocessing and that appear more than 10% in the dataset to generate association rules. As mentioned above, association rules are logical expressions that are represented through sentences with the structure:

(2) Ant→Con

(3) $${\text{item}}_{a},\ldots ,{\text{item}}_{n}\to {\text{item}}_{r},\ldots ,{\text{item}}_{u}$$

Since the items in Ant are not found in Con, then Ant∩Con=∅. To evaluate the relevance of the obtained association rules, the following metrics are used:

(4) support=P(Ant∩Con)

(5) $$\text{confidence}=\frac{P\left(\mathrm{Ant}\cap \mathrm{Con}\right)}{P\left(\mathrm{Ant}\right)}$$

(6) $$\text{lift}=\frac{P\left(\text{Ant}\cap \mathrm{Con}\right)}{P\left(\mathrm{Ant}\right)\cdot P\left(\mathrm{Con}\right)}$$

(7) $$\text{bi‐lift}=\frac{P\left(\text{Ant}\cap \mathrm{Con}\right)P\left(\bar{\mathrm{Ant}}\right)}{P(\bar{\mathrm{Ant}\cap \mathrm{Con}})P\left(\mathrm{Ant}\right)}$$

(8) $$\text{odds ratio}=\frac{P\left(\mathrm{Ant}\cap \mathrm{Con}\right)P\left(\bar{\mathrm{Ant}\cap \mathrm{Con}}\right)}{P\left(\bar{\mathrm{Ant}}\cap \mathrm{Con}\right)P\left(\mathrm{Ant}\cap \bar{\mathrm{Con}}\right)}$$

where *P*(X) with X = Ant, Con is the probability that X happens, $P\left(\mathrm{Ant}\cap \mathrm{Con}\right)$ is the probability of Ant and Con happening; $P(\bar{\mathrm{Ant}}\cap \mathrm{Con})$ is the probability that Ant is no happening and Con occurring; $P(\mathrm{Ant}\cap \bar{\mathrm{Con}})$ is the probability that Con is no happening and Ant takes place; $P(\bar{\mathrm{Ant}\cap \mathrm{Con}})$ is the probability that neither Ant nor Con comes to pass. It is worth noting that support measures the portion of data that satisfies the rule. The confidence corresponds to the conditional probability, while the lift measures the independence between antecedent and consequent. Usually, these three metrics are considered to evaluate the association rules. However, these metrics do not provide information about the correlation between antecedent and consequent. For this reason, it is considered the odds ratio metric which measures correlation [25]. So, the further away from 1 in the odds ratio, the greater the relationship between antecedents and consequents [26]. Additionally, it is considered the bi‐lift metric [27], to distinguish between Ant → Con and Con → Ant, which cannot be evaluated with the other metrics [28]. While some rules may involve identical items, their distribution between Ant and Con can lead to a set of rules with varying metric values. In this case, we selected the rule with the highest lift value.

In this way, 72, 690 association rules are generated. For analyzing these rules, we focus on the bandgap and the features that may be associated with it. These features are identified through a matrix correlation analysis. Thus, the main descriptors considered are *E*_g_, *E*_gc_, Chain, dNHal, Dis, InterL, Ang180, LSF1, and LSF2. For each of these descriptors, a subset grouping the rules containing it is formed. If one of these subsets includes more than 100 rules, an agglomerative clustering [29, 30] is applied as it is explained in Supporting Information. For each cluster, the maximum value of the accumulative metric is identified, allowing us to determine the cluster with the best metric for further analysis. By accumulative metrics, we mean the addition of all the metric values for a given rule, considering the metric values standardized to range from zero to one. The extraction of association rules, the calculation of the specified metrics, and the identification of relevant rules are performed using a Python script included in the Supporting Information. The best rules are analyzed through graph plots created using Gephi software [31, 32].

## 3 Results

**FIGURE 5:**
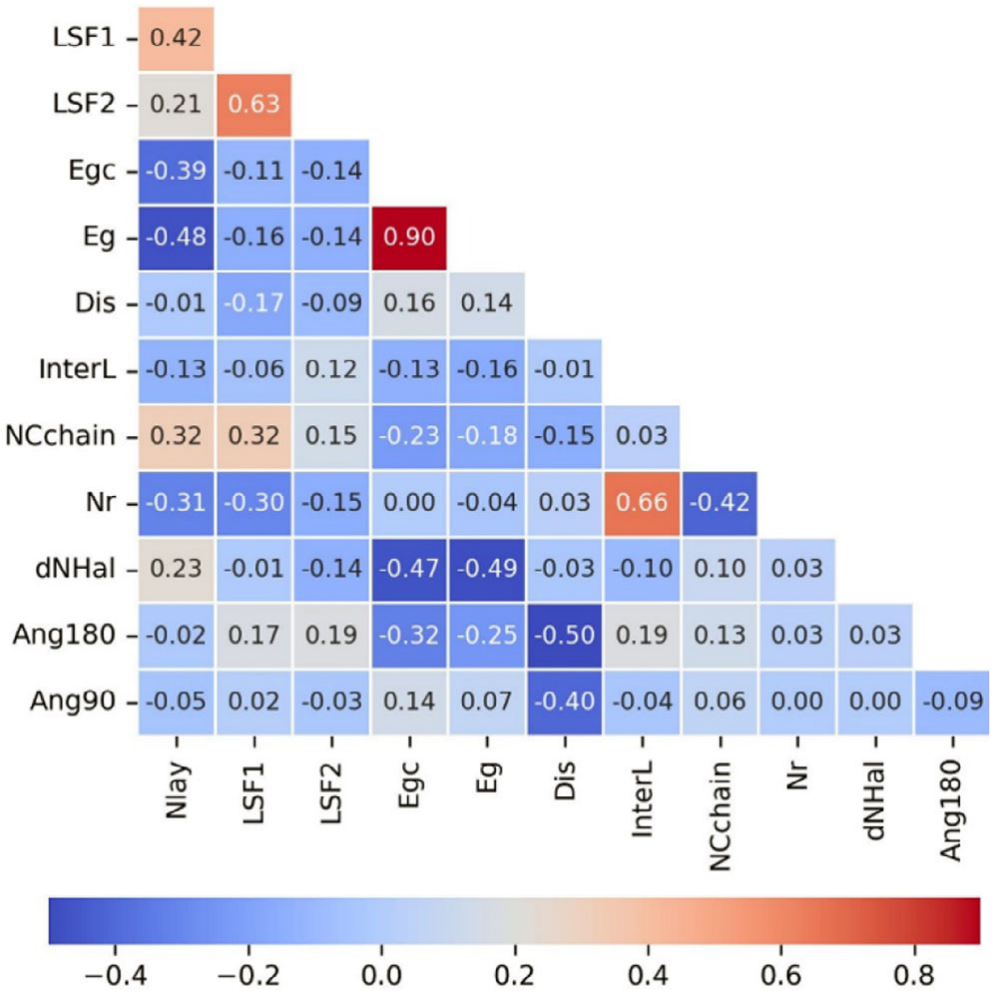
Heat map of the correlation matrix involving the predictors of interest.

First, the relationship between numerical predictors in the dataset is analyzed using a correlation matrix, which is visually represented as a heat map (Figure 5), where red represents a correlation of one, blue a correlation of minus one, and white zero correlation. Notably, the largest negative correlations of *E*_g_ are with NLay and dNHal, meaning that as NLay and dNHal increase, *E*_g_ decreases.

In terms of the properties of organic layers, there is a positive correlation between InterL and the number of carbon rings in the spacer organic cation (Nr), as one may expect. This relationship is due to the size increment of the organic cation as the number of carbon rings rises. Moreover, Dis negatively correlates with Ang90 and Ang180, which is consistent since the smaller the angle value, the greater the deformation in the octahedral structures of perovskites. These factors may influence *E*_g_ and *E*_gc_ due to their negative correlation with Ang180, which is also related to the characteristics of organic cations, as previously mentioned.

Table 4 shows the 23 association rules (A.1–A.23) that contain *E*_g_. It is worth noting that the support for all rules is above 0.1, and the maximum support of these rules is 0.17, which implies that the rules are satisfied between 10% and 17% in the dataset. Although this could be seen as a low value, one should consider that the *E*_g_ item with the highest frequency is *E*_g_|2, which has a maximum probability of 0.22. Considering solely the support metric may not be enough to identify the relevance of the rules effectively because support is determined by the probability that a rule holds true, and it does not consider the correlation or relationship between Ant and Con; e.g., A.1 has a support value of 0.11 as many other rules but a confidence of 1, which means that the conditional probability is 100%, so that if the material exhibits the items *A*|1 and *E*_g_|5, then also present *C*|1. Thus, the A.1 rule outlines the relationship between the cell parameters *a* and *c*, which have values less than 8.7 Å and the bandgap values exceeding 2.79 eV (*E*_g_|5). It is worth mentioning that there is no association rule linking cell parameter *b* to *E*_g_. Likely the latter is due to a nondirect correlation between the *b* and *E*_g_ values, as shown in Figure S3, Supporting Information. Another example in which support does not identify the relevance of the rule is for the case of A.2 (Table 4). A.2 contains items like those in A.3 and A.4 but with a support value smaller than those for A.3 and A.4. However, A.2 is the most representative of these three rules because it has a higher value of lift and confidence. Moreover, A.2 encompasses A.3 and A.4 and involves the relationship between *E*_g_|5, dNHal|1, and *E*_gc_|5, which agrees with the information obtained from the exploratory analysis of the dataset, where the high values of *E*_g_ correspond to small values of dNHal.

**TABLE 4:** Relevant association rules corresponding to *E*_g_ descriptor.

Rule	Ant	Con	Conf	Supp	Lift
A.1	*A*|1, *E*_g_|5	*C*|1	1.00	0.11	5.03
A.2	*E*_g_|5, dNHal|1	*E*_gc_|5	0.96	0.12	4.66
A.3	*E*_g_|5	*E*_gc_|5	0.83	0.17	4.05
A.4	dNHal|1	*E*_g_|5	0.67	0.13	3.26
A.5	*E*_g_|1	*E*_gc_|1	0.62	0.11	3.12
A.6	*E*_g_|1	Cinter| MethylAm	0.66	0.11	2.80
A.7	*E*_g_|5	*C*|1	0.54	0.11	2.73
A.8	Hal|Br	*E*_g_|5	0.55	0.14	2.68
A.9	*E*_g_|5	*A*|1	0.51	0.11	2.59
A.10	*E*_g_|4	*E*_gc_|4	0.51	0.11	2.31
A.11	Chain|Arom, *E*_g_|3	NCr|6	0.72	0.11	2.28
A.12	Chain|Arom, *E*_g_|3	Nr|1	0.88	0.13	2.21
A.13	Nr|1	*E*_g_|3	0.35	0.14	1.78
A.14	NCr|6	*E*_g_|3	0.35	0.11	1.77
A.15	*E*_g_|3	Chain|Arom	0.74	0.15	1.52
A.16	LSF2|4	*E*_g_|2	0.28	0.12	1.25
A.17	*E*_g_|1	Chain|Ali	0.62	0.11	1.21
A.18	*E*_g_|5	Chain|Ali	0.60	0.12	1.17
A.19	*E*_g_|2	Chain|Ali	0.58	0.13	1.13
A.20	*E*_g_|4	Beta|2	0.57	0.12	1.12
A.21	*E*_g_|2	Beta|2	0.55	0.12	1.09
A.22	*E*_g_|4	Chain|Ali	0.51	0.11	1.00

Conf corresponds to confidence and the supp to support.

Some of the rules like A.3, A.5, and A.10 solely connect *E*_g_ and *E*_gc_ items. According to the lift measure, *E*_g_|5 and *E*_gc_|5 are significantly related, followed by *E*_g_|1 and *E*_gc_|1, and finally by *E*_g_|4 and *E*_gc_|4. This behavior is in concordance with the trend observed in the dispersion plot of *E*_gc_ versus *E*_g_ (Figure S1).

The A.21 and A.22 involve the angle beta (beta|2) in the range from 90° to 93° with *E*_g_|2 and *E*_g_|4, respectively. These rules exhibit similar support to the other rules in Table 4; however, their confidence and lift values are low, therefore are not further considered. The symmetry of the materials in the dataset is also considered in the analysis. Nevertheless, no association rules linking the *E*_g_ to the space groups are found (see Table S2 and Figure S4, Supporting Information).

**FIGURE 6:**
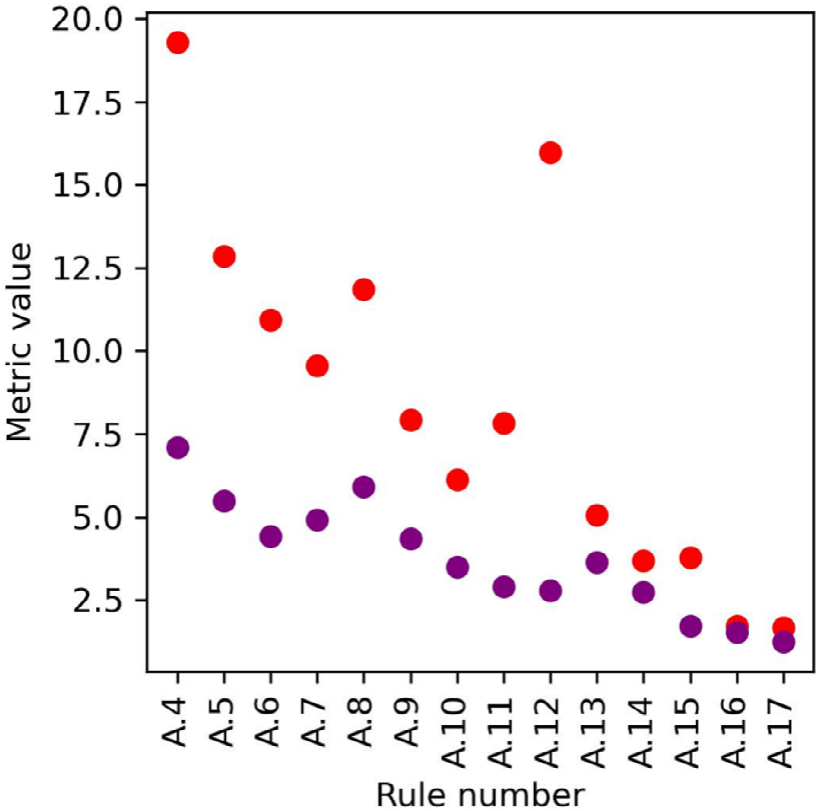
Odds ratio and bi‐lift values for some of the association rules. The purple dots represent the odds ratio metric and red dots to bi‐lift.

Correlation between Ant and Con is evaluated using the odds ratio metric, while the independence of events with respect to the order Ant and Con is assessed with bi‐lift, as mentioned above. Both measures show a similar trend but with the odds ratio given larger values (Figure 6). Also important to notice is that odds ratio and bi‐lift exhibit significant variations among the rules, providing a useful tool to distinguish the best ones as compared to other metrics such as support, confidence, and lift. A.4, A.12, A.5, and A.8 have the four largest odds ratio values. A.4 involves dNHal|1 and *E*_g_|5 which agrees with the negative correlation between dNHal and *E*_g_, as mentioned above. However, A.5, the third largest odd ratio value, denotes correlation between *E*_g_|1 and *E*_gc_|1, which is somewhat an expected result. The large odds ratio value for A.12, which is the second largest, may be due to the strong relationship between aromatic compounds (Chain|Arom) and their characteristic of having a single ring (Nr|1) for materials with Eg|3. In contrast, A.8 demonstrates a significant correlation between high bandgap values (*E*_g_|5) and the bromine presence in the octahedral structure of perovskites.

**FIGURE 7:**
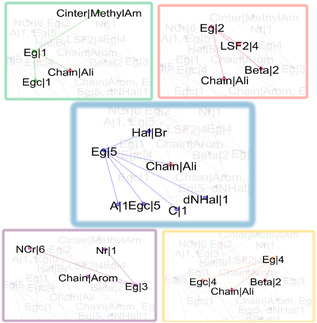
Relevant association rules involving *E*_g_. Each box corresponds to one of the *E*_g_ items: green box *E*_g_|1, red box *E*_g_|2, purple box *E*_g_|3, yellow box *E*_g_|4, and blue box *E*_g_|5.

To summarize graphically what is mentioned above, Figure 7 shows links between the primary items related to *E*_g_ and each of the *E*_g_ items. In this way, one notices that *E*_g_|1, *E*_g_|2, *E*_g_|4, *E*_g_|5 are linked with Chain|Ali and only *E*_g_|3 is connected with Chain|Arom. Moreover, one finds that *E*_gc_ is associated with *E*_g_ for quintiles 1, 4 and 5, which is a consistent result noticed above. Yet, no rule correlates *E*_gc_|2 with *E*_g_|2 nor *E*_gc_|3 with *E*_g_|3, suggesting that the machine learning model used in ref. [15] is not adequate to predict bandgaps in quintiles 2 and 3, which is also illustrated by the large dispersion of data in plot S1 (see Supporting Information). It is worth mentioning that around 30% of rules with *E*_g_ correspond to values in the fifth quintile (*E*_g_|5), which range from 2.79 to 5.29 eV. Among the relevant properties associated with *E*_g_|5 is dNHal|1, which ranges from 2.96 to 3.49 Å.

**FIGURE 8:**
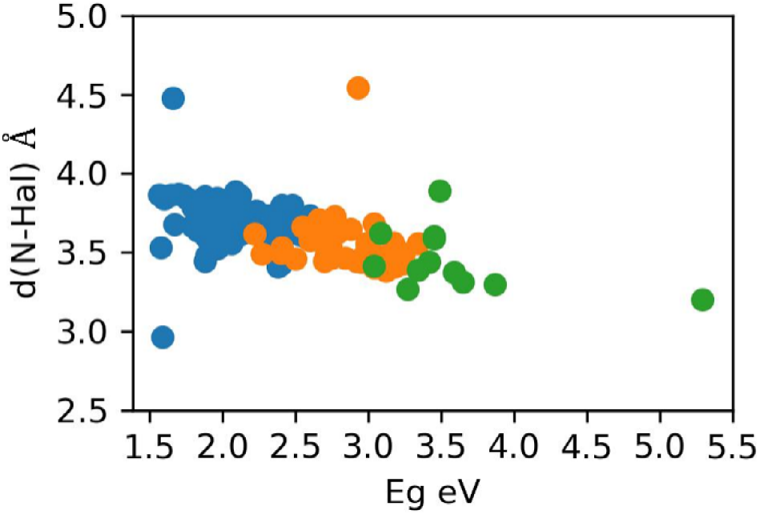
Bandgap values and the distance N–Hal for the investigated perovskites. The blue dots correspond to material that contains iodine as halogen, orange dots to bromine, and green to chlorine.

Figure 8 shows the trend of *E*_g_ increasing while the distance N–Hal decreases, which agrees with the information obtained from association rules and the correlation matrix (Figure 5). In this way, this negative correlation between *E*_g_ and dNHal is more pronounced in materials with iodine and bromine. Hence, the halogen type affects *E*_g_; materials with iodine tend to have a lower value than those with bromine, while the largest *E*_g_ values correspond to materials with chlorine. Thus, roughly speaking, the larger the electronegativity of the halogen, the larger the bandgap of the material. The atypical values of dNHal in Figure 8 correspond to materials with organic spacers, such as octanoyldiamonium, or aromatic compounds that contain many carbon rings. Therefore, these atypical values may be attributed to steric effects connected to the size of the organic spacers. Specifically, certain aromatic compounds increase dNHal, and the large aliphatic chains reduce it [11]. The nature of the influence of dNHal on the bandgap values may be due to the electrostatic interaction between the positive charge of N and the negative one of the halogen. It was reported that this interaction could affect the properties and structure of 2D perovskites [10].

Table 5 also presents rules that link items from *E*_gc_ and Chain, with the latter containing only two types of items (Chain|Ali and Chain|Arom). Rules B.34 and B.35 indicate that *E*_gc_|4 has a relationship with either Chain|Ali or Chain|Arom, both demonstrating a confidence level of 0.5. This confidence suggests that materials containing *E*_gc_|4 may include either aliphatic or aromatic interlayer cations. Likewise, rule A.22 indicates that *E*_g_|4 can have aliphatic interlayer cations, with a conditional probability of 51%. A similar pattern is observed for *E*_g_|2 and *E*_gc_|2, as shown in rules A.19 and B.33, respectively. However, materials with *E*_g_|1, *E*_g_|3, and *E*_g_|5 are associated with only one type of interlayer cation.

**TABLE 5:** Relevant association rules.

Rule	Ant	Con	Conf	Lift
B.1	*A*|2, LSF2|4	*C*|2	1.00	5.18
B.2	*E*_gc_|1, Phase|RP	Chain|Ali, LSF1|5	0.91	4.99
B.3	NCchain|4, Phase|RP	Chain|Ali, LSF1|5, LSF2|4	0.86	4.73
B.4	*E*_gc_|1, LSF2|4, Phase|RP	LSF1|5	0.91	4.30
B.5	Chain|Ali, LSF2|4, Phase|RP	LSF1|5	0.84	3.98
B.6	Chain|Ali, InterL|3, LSF2|4	Phase|RP	0.90	3.50
B.7	Ang180|3, Chain|Ali	Cinter|MethylAm	0.78	3.35
B.8	NCchain|4	InterL|3	0.60	3.02
B.9	Dis|5	Ang180|1	0.57	2.87
B.10	*E*_gc_|1	Ang180|3	0.56	2.81
B.11	NCchain|2, NCr|6	Ang180|4, Chain|Arom	0.62	2.79
B.12	Ang180|4, Chain|Arom	InterL|5	0.53	2.57
B.13	Ang180|4, Phase|RP	LSF2|4	1.00	2.38
B.14	Dis|1	Ang180|4	0.91	2.26
B.15	Chain|Arom, LSF2|4	Ang180|4	0.78	1.93
B.16	Ang180|4, Beta|2	LSF2|4	0.79	1.88
B.17	InterL|5	Chain|Arom	0.83	1.71
B.18	InterL|5	Ang180|4	0.69	1.70
B.19	Dis|2	Ang180|4	0.68	1.68
B.20	LSF1|5	Chain|Ali	0.86	1.67
B.21	*E*_gc_|3	Ang180|4	0.63	1.57
B.22	dNHal|3	Chain|Arom	0.76	1.56
B.23	LSF1|2	Chain|Arom	0.74	1.53
B.24	InterL|2	Chain|Ali	0.77	1.49
B.25	InterL|3	Chain|Ali	0.77	1.49
B.26	dNHal|5	Chain|Ali	0.71	1.39
B.27	LSF1|4	Ang180|4	0.54	1.35
B.28	dNHal|2	Ang180|4	0.54	1.35
B.29	InterL|4	Chain|Arom	0.62	1.27
B.30	LSF2|2	Chain|Arom	0.61	1.26
B.31	dNHal|1	Chain|Ali	0.64	1.24
B.32	*E*_gc_|3	Chain|Arom	0.60	1.24
B.33	*E*_gc_|2	Chain|Arom	0.56	1.15
B.34	*E*_gc_|4	Chain|Arom	0.50	1.03
B.35	*E*_gc_|4	Chain|Ali	0.50	0.97

Conf stands for confidence and supp for support.

**FIGURE 9:**
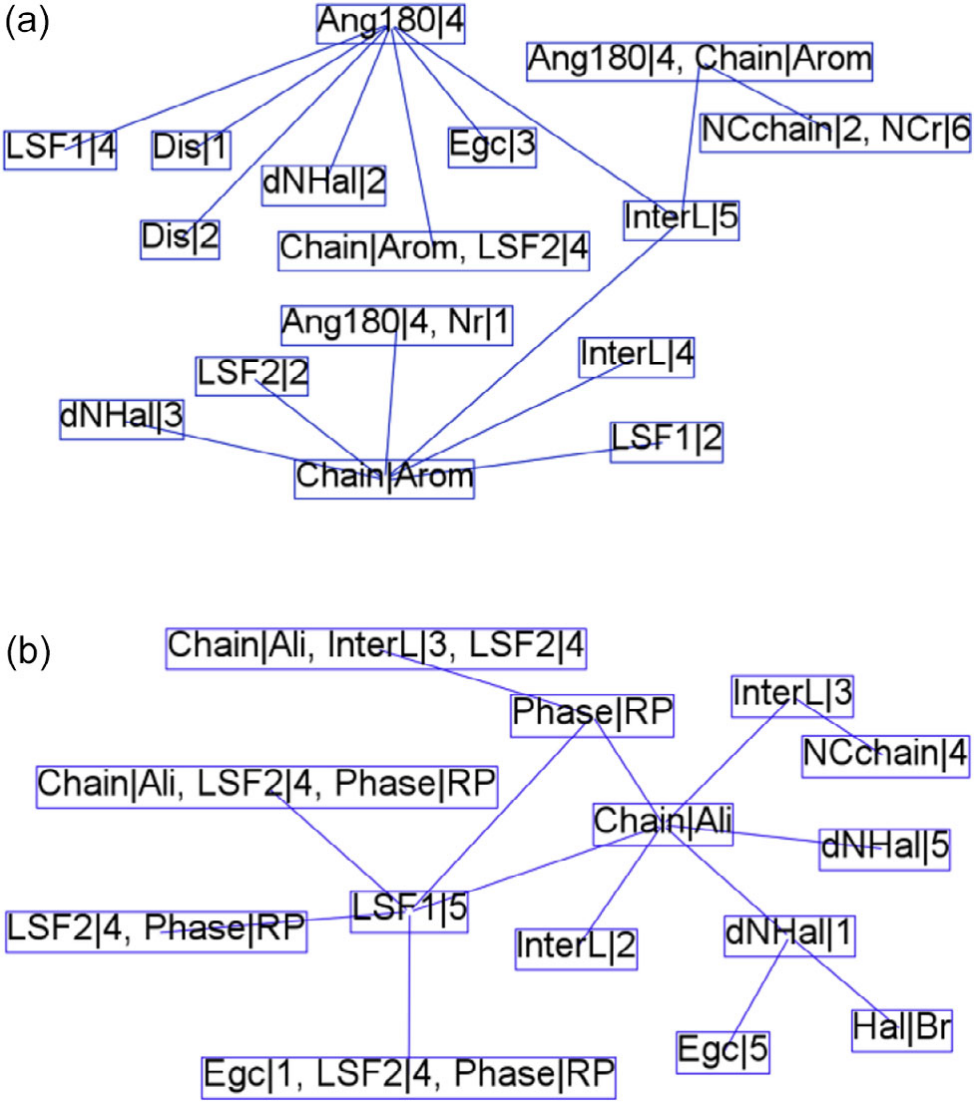
Link among items (a) corresponding to association rules that contain Chain|Arom and (b) corresponding to association rules that contain Chain|Ali.

Specifically, materials with *E*_g_|1 and *E*_g_|5 are linked to aliphatic compounds, while *E*_g_|3 is associated with aromatic cations that usually feature a single phenyl ring, as stated in rules A.11 and A.12. Inspecting the items corresponding to association rules containing Chain|Arom (Figure 9a), it is found that Ang180|4 is a recurrent item closely associated with aromatic interlayer cations that have a phenyl group substituted by methylammonium (rule B.11).

Consequently, these aromatic compounds cause only a slight deviation in the axial angles of the octahedral structure of the inorganic layer, which is consistent with the low distortion values (Dis1 and Dis2) observed for these structures. Thus, the aromatic cations induce rigid structures, limiting their influence on the geometrical characteristics of the inorganic layers.

2D‐hybrid perovskites with aromatic spacer cations exhibit large interlayer distances (InterL|5 and InterL|4), likely due to the considerable size of these organic compounds. Layer shift was analyzed through the descriptors LSF, which demonstrate an intermediate shift represented by LSF1|2 (0.15–0.22) and LSF2|2 (0.25–0.39) for the mentioned materials. Consequently, hybrid perovskites with aromatic interlayer cations exhibit shifts between inorganic layers, indicating that they do not fall under phase DJ (LSF(0,0)). However, the observed shift is not significant enough to categorize them as phase RP (LSF(0,1/2) or LSF(1/2,1/2)). In this case, evaluating the LSF predictor is a helpful tool that enables the representation of the shift layer of material beyond the classifications DJ or RP.

As shown in Figure 9b, the 2D‐hybrid perovskite with aliphatic interlayer cation (Chain|Ali) usually presents a phase RP and LSF1 and LSF2 around 0.5 (LSF1|5 and LSF2|4). Moreover, the interlayer distances (InterL|3 and InterL|2) are shorter than the aromatic compounds. According to rule B.8, which has a high lift value that denotes a representative relationship between the aliphatic interlayer cations with a chain of four carbon atoms (NCchain|4)), such as butylammonium, and to interlayer distance InterL|3.

**FIGURE 10:**
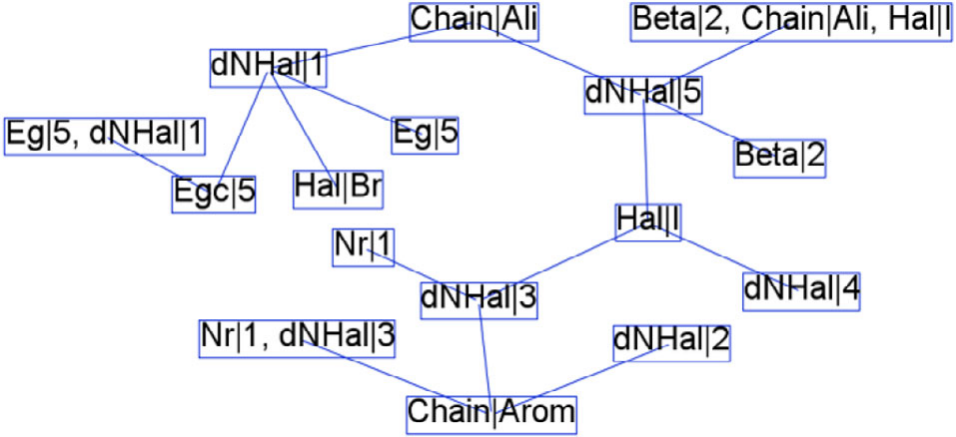
Link among items of association rules corresponding to distance N–Hal (Table S3).

It is worth mentioning that the distance N–Hal is related not only to the kind of halogen but also to the type of organic cation, whether aliphatic or aromatic. As shown in Figure 10, the materials with aromatic cations often exhibit distance N–Hal intermediates of the second to fourth quintile (dNHal|2, dNHal|3, dNHal|4) with iodine as halogen. Meanwhile, the materials with aliphatic cations have short N–Hal distances (dNHal|1) when the halogen is bromine and corresponds to high bandgap values (*E*_g_|5). However, if the halogen is iodine and the interlayer cation is aliphatic, the distance N–Hal will be large (dNHal|5), and the materials with these characteristics could exhibit a low calculated bandgap (*E*_gc_|1). This can be interpreted in terms of electronegativity, as bromine is more electronegative than iodine. Therefore, the interaction between bromine and the aliphatic cation is significantly stronger than that of iodine with this cation.

## 4 Discussion

The data mining analysis of 2D‐hybrid halide perovskites identifies key predictors that represent essential aspects of these materials and their interrelationships with the bandgap. One significant descriptor is the N–Hal distance, which characterizes the interaction between the inorganic layer and the organic spacer cation. The inorganic layer and the organic spacer are represented by the descriptors Ang180 and Chain, respectively. The structure of these materials is defined by descriptors such as the interlayer distance, the halogen in the inorganic layer, the RP phase, and the LSF factor.

Correlation analysis and the association rules show a close relationship between the N–Hal distance and the bandgap, the values for the latter increase while N–Hal distance decreases. This trend is influenced by other properties, such as the halogen in the perovskite structure and whether the organic cation is aliphatic or aromatic. Hence, the kind of halogen influences the bandgap, a result that agrees with other studies [15].

Importantly, this analysis reveals patterns that connect the chemical properties of the organic spacer cation with the bandgap. For perovskites that contain iodine, the presence of aliphatic cations correlates with small bandgap values, from 1.57 to 1.93 eV. In comparison, those that contain aromatic cations are associated with bandgap values between 2.18 and 2.38 eV. Conversely, 2D‐hybrid perovskites with bromine exhibit large bandgap values, ranging from 2.79 to 5.29 eV, and short N–Hal distances, with values in between 2.96 and 3.49 Å.

The chemical nature of the organic spacer affects the distortion of the octahedral inorganic structure, the interlayer distance, the phase, and the shift layer. Aliphatic compounds are linked to the RP phase, while aromatic compounds are associated with intermediate LSF values, i. e., inducing a phase that is neither RP nor DJ.

Patterns observed in this study emphasize the interaction between the inorganic and organic layers, highlighting their significance for bandgap and structural properties. This information could be essential for designing new materials by optimizing the composition of both inorganic and organic layers.

## 5 Conclusion

The present contribution explores the extraction of knowledge from 2D‐hybrid halide perovskites by data mining techniques. In this way, the association rules identify the key relationship between the bandgap and the structural and composition properties of these materials.

The N–Hal distance is identified as a relevant descriptor, as it decreases with increasing bandgap. This descriptor is associated with the kind of halogen present in the inorganic layer and the organic cation spacer. Among the structural descriptors, the distortion and the angles in the octahedral that conform to the inorganic layers, as well as the LSF, are highlighted.

The obtained results reveal the importance of the chemical nature of spacer cations, which influence the structural parameter and the bandgap. The 2D‐hybrid halides with aliphatic cations and iodine as halogens are associated with a small bandgaps and large values when the halogen is bromine. Intermediate bandgaps are frequently linked to materials that contain aromatic cation spacers.

The identified patterns and associations help in gaining new knowledge about these semiconductors, which may be crucial for optimizing and designing new materials with improved properties for applications in the energy and electronic devices sectors.

## Supporting Information

Additional supporting information can be found online in the Supporting Information section. The supporting information includes the features considered, preprocessing of numeric data, selection of relevant association rules, the metric values corresponding to the distance N‐Hal, and Python code to obtain the association rules. **Supporting Fig. S1:** Dispersion plot of experimental band gap (Eg) vs calculated band gap (Egc). The purple dotted lines indicate the range for each quintile of experimental band gap (from Eg|1 to Eg|5) and calculated band gap (Egc|1 to Egc|5). Calculated band gaps correspond to those reported by Marchenko et al. who utilized machine learning techniques for estimation [15]. **Supporting Fig. S2:** Diagram illustrating the stage of selecting relevant rules. **Supporting Fig. S3:** Dispersion plot of experimental band gap and b cell parameters. **Supporting Fig. S4:** Bar plot of the frequency of representative space groups for each quintile of the experimental band gap from Eg|1 to Eg|5. **Supporting Table S1:** Intervals and items used in the dataset. **Supporting Table S2:** Symmetry space groups of 2D hybrid perovskites and their frequencies in the dataset. **Supporting Table S3:** Relevant association rules corresponding to distance N‐Hal.

## Funding

The authors have nothing to report.

## Conflicts of Interest

The authors declare no conflicts of interest.

## Supporting information

Supplementary Material

## Data Availability

The data that support the findings of this study are available in the supplementary material of this article.
