# Supplementary material for: Extracting Knowledge of 2D‐Hybrid Halide Perovskite Materials: A Data Mining Approach
Source: ChemistryOpen. 2026 Mar 12;15(3):e202500606. doi: 10.1002/open.202500606 (PMC13097588; doi:10.1002/open.202500606)
Supplement: Supplementary file 1 — Supplementary Material [file OPEN-15-e202500606-s001.pdf]

Supporting Information: Extracting knowledge of 2D-hybrid halide perovskite materials, A Data Mining Approach.

Guadalupe Castro, Miriam Pescador-Rojas and Joel Ireta

Dataset

The data used in this contribution is sourced from the reports by Marchenko et al.[15] We extract data from the crystallographic information framework file of 171 materials to obtain 28 descriptors, which are listed in Table S1.

Table S1. Descriptors for 2D hybrid perovskites used to obtain association rules.

Descriptor	Description
A	A cell parameter
alpha	Angle alpha cell parameter
Ang180	Mean of angles of octahedral near to 180 (axial)
Ang90	Mean of angles of octahedral near to 90 (ecuatorial)
B	B cell parameter
beta	Angle beta cell parameter
C	C cell parameter
Chain	Chain organic type (aliphatic or aromatic)
Cinter	Cation intralayer
Dis	Octahedral distortion
dNHal	Distance means of N terminal to Halogen
Eg	Experimental band gaps
Egc	Calculated band gaps
Ginter	Functional group in the organic cation interlayer
Hal	Halogen
InterL	Distance interlayer
LSF1	Layer shift factor-translation 1 (shift octahedral layers)
LSF2	Layer shift factor-translation 2 (shift octahedral layers)
M	Metalic center
NCC	Carbons number in the aliphatic chain
NCr	Carbons number in the aromatic ring
Nlayer	Layer number
NN	Number of nitrogen terminals in chain organic
Nr	Number of rings in the organic compound
Phase	Ruddlesden-Popper (RP) or Dion-Jacobson (DJ) phase
Qc	Formal charge of organic cation
S2D	2D exposed plane
Sym	Symmetry

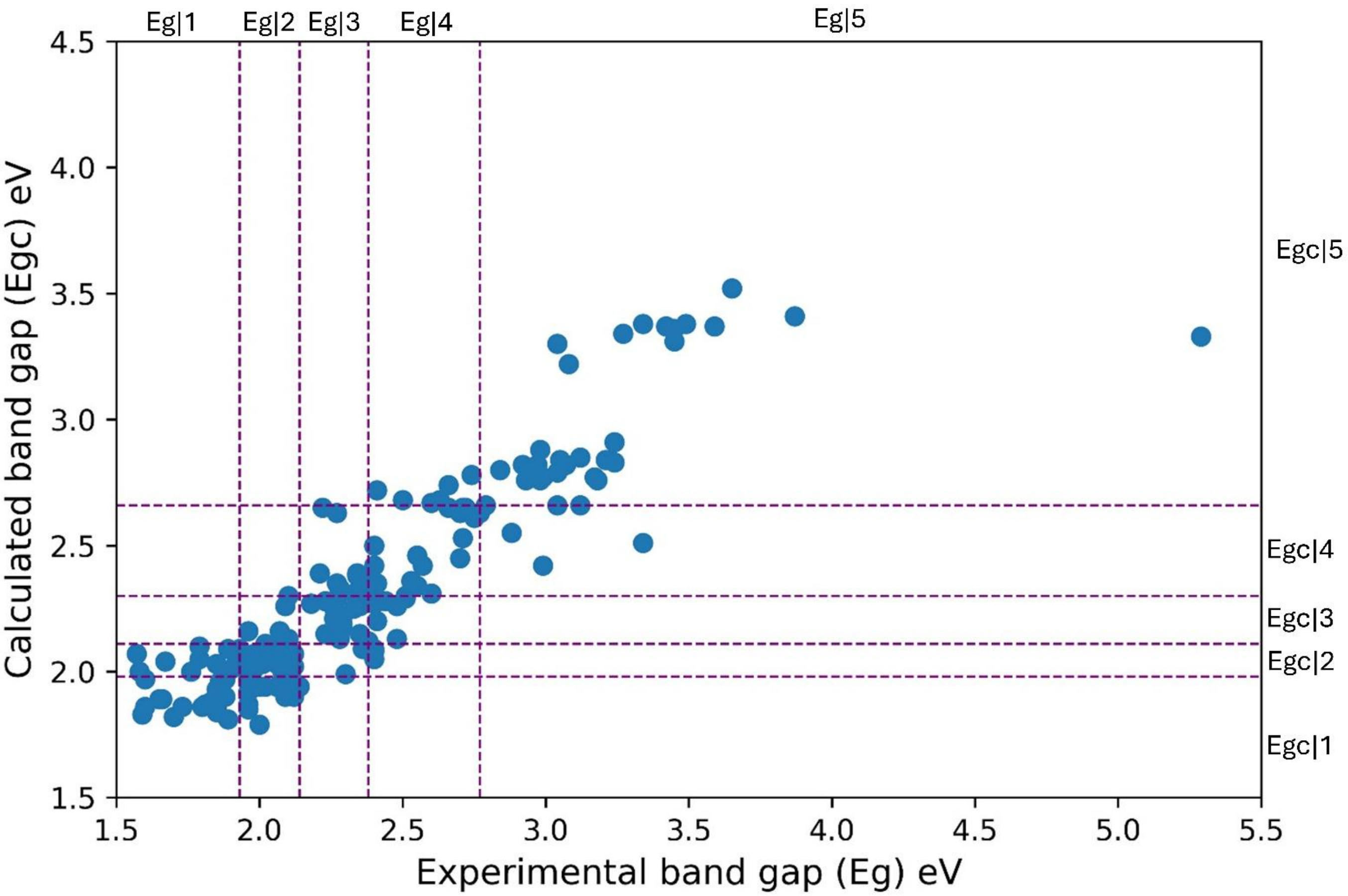

Figure S1. Dispersion plot of experimental band gap (Eg) vs calculated band gap (Egc). The purple dotted lines indicate the range for each quintile of experimental band gap (from Eg|1 to Eg|5) and calculated band gap (Egc|1 to Egc|5). Calculated band gaps correspond to those reported by Marchenko et al. who utilized machine learning techniques for estimation.[15]

Preprocessing of numeric data.

As part of data preprocessing, the numeric data is discretized into quintiles to create items with similar frequency. The label for each item and its corresponding interval is listed in Table S1.

Table S1. Intervals and items used in the dataset

Item	Interval	Item	Interval
A|1	[5.764,8.686]	dNHal|1	[2.964,3.489]
A|2	[8.696,9.122]	dNHal|2	[3.496,3.621]
A|3	[9.156,16.759]	dNHal|3	[3.623,3.674]
A|4	[16.813,35.07]	dNHal|4	[3.675,3.735]
A|5	[35.769,74.599]	dNHal|5	[3.737,4.546]
Alpha|1	[78.96,88.33]	Eg|1	[1.57,1.93]
Alpha|2	[90.0,101.1]	Eg|2	[1.96,2.14]
Ang180|1	[148.969,173.917]	Eg|3	[2.18,2.38]
Ang180|2	[173.973,176.081]	Eg|4	[2.39,2.77]
Ang180|3	[176.126,178.332]	Eg|5	[2.79,5.29]
Ang180|4	[178.459,180.0]	Egc|1	[1.79,1.98]
Ang90|1	[88.72,89.969]	Egc|2	[1.99,2.11]
Ang90|2	[89.97,89.993]	Egc |3	[2.12,2.3]
Ang90|3	[89.995,89.999]	Egc|4	[2.31,2.66]
Ang90|4	[90.0,91.865]	Egc|5	[2.67,3.52]
B|1	[5.945,8.378]	InterL|1	[2.6,5.19224]
B|2	[8.383,8.796]	InterL|2	[5.1985,7.106762]
B|3	[8.85,8.976]	InterL|3	[7.1123,8.132161]
B|4	[8.993,12.601]	InterL|4	[8.1758,10.410981]
B|5	[12.927,101.959]	InterL|5	[10.4585,30.98175]
Beta|1	[69.57,85.28]	LSF1|1	[0.01,0.14]
Beta|2	[90.0,93.0]	LSF1|2	[0.15,0.22]
Beta|3	[93.04,97.6]	LSF1|3	[0.23,0.38]
Beta|4	[97.62,127.65]	LSF1|4	[0.39,0.48]
C|1	[5.764,8.681]	LSF1|5	[0.49,0.5]
C|2	[8.686,9.122]	LSF2|1	[0.02,0.24]
C|3	[9.156,16.813]	LSF2|2	[0.25,0.39]
C|4	[16.972,35.07]	LSF2|3	[0.4,0.47]
C|5	[35.769,68.752]	LSF2|4	[0.48,0.5]
Dist|1	[0.0,2.7e-05]		
Dist|2	[0.0,7.9e-05]		
Dist|3	[0.0001,0.000273]		
Dist|4	[0.0003,0.000631]		
Dist|5	[0.0006,0.031794]		

Selection of relevant association rules

Due to the extensive number of association rules, it is essential to define the properties of interest for 2D hybrid perovskites. These properties include the band gap, type of organic cation chain, N-Hal distance, distortion, interlayer distance, the angle between axial atoms in the octahedral structure, and the layer shift factor. Rules containing high-frequency items are removed, as they can lead to biased or overly simplistic interpretations.

The stages for selecting relevant association rules are outlined in Figure 2S. First, a set of rules that pertains to a single property of interest is chosen. We proceed to the second stage if this set contains more than fifty rules. Here, five metric values (support, confidence, lift, bi-lift, and odds ratio) are standardized to range between zero and one. In the third-stage, agglomerative clustering is applied to the dataset, resulting in rules clustered based on their metrics.

Agglomerative clustering is a method that forms clusters using a bottom-up approach, starting with n clusters, each containing a single element. The clusters are then merged based on a distance measurement through an iterative algorithm. This process continues until all elements are consolidated into a single cluster. In this contribution, the Ward's method is employed to determine how clusters are linked. This approach minimizes the variance by considering both the distance between the elements of each cluster and the distances between different clusters. It is worth mentioning that the Ward's method uses Euclidean distance. The number of clusters was chosen by considering the clusters formed with a distance less than 0.7 times the maximum distance between all clusters.

In the fourth stage, we analyze the metric values for each cluster and select the one with the most favorable metrics. Finally, we analyze and interpret the rules from the chosen cluster.

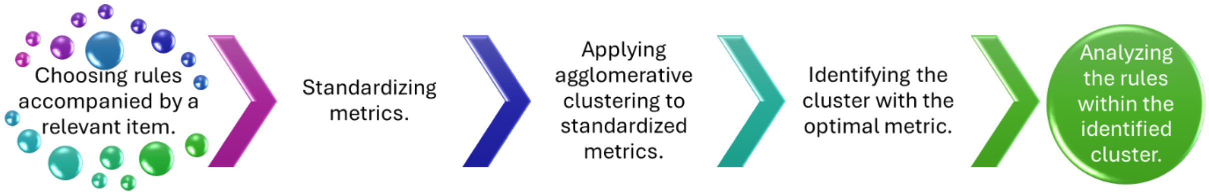

Figure 2S. Diagram illustrating the stage of selecting relevant rules.

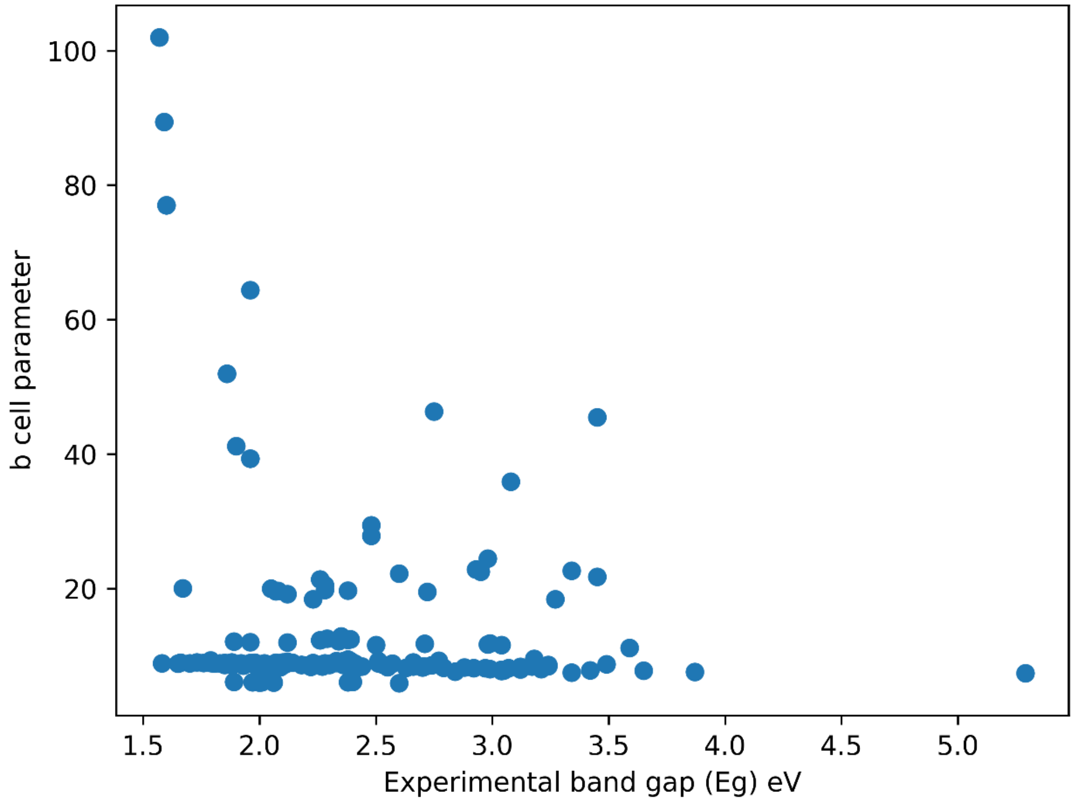

Figure 3S. Dispersion plot of experimental band gap and b cell parameters.

Table 2S. Symmetry space groups of 2D hybrid perovskites and their frequencies in the dataset.

Space Group	Frequency
$P2_1/c$	34
Pbca	18
Cc	17
$P\bar{1}$	17
$Cmc2_1$	13
C2/c	12
Pc	7
Pnma	5
Cmcm	5
P1	4
Ama2	3
Cmca	3
C2/m	3
Cmca (Acam)	2
Aba2 (C2cb)	2
Aba2	2
$Pna2_1$	2
$P2_1$	2
Pbcn	2
$P2_1/c$ ($P2_1/n$)	2
Cmcm (Cccm)	1
Cmcm (Acam)	1
Aba2(C2cb)	1
$Cmc2_1$ ($A2_1ma$)	1
$P2_12_12_1$	1
C2	1
Pccn	1
$P4_2/ncm$	1
$P2_1c$	1
P42 c m	1
Pmna	1
C/2c	1
$P2_1/c$ ($2_1/n$)	1
$P2_1/c$ ($P2_1/a$)	1
Pc (Pn)	1
$P2_1/m$	1

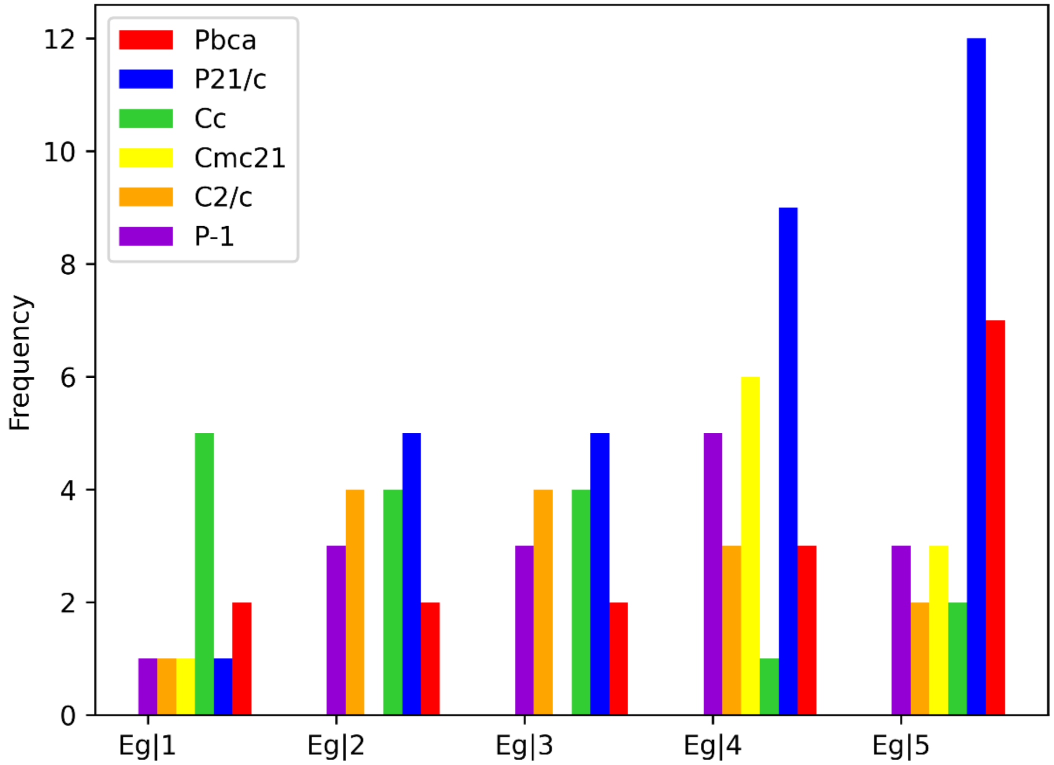

Figure 4S. Bar plot of the frequency of representative space groups for each quintile of the experimental band gap from Eg|1 to Eg|5.

The dataset includes a wide range of space groups, showcasing the diversity of materials and their structural properties. Table 2S shows that among the 36 space groups, only 6 have frequencies greater than 10. This means that in the dataset, more than 10 materials exhibit one of these space groups. Figure 4S shows the frequency of these six space groups (P21/c, Pbca, Cc, P-1, Cmc21, and C2/c) with respect to the quintiles of Eg (from Eg|1 to Eg|5). Highlighting that in Eg|1, the majority of materials have space group Cc. The frequency of this group decreased from Eg|1 to Eg|5. The number of materials with symmetry P21/c, however, increases from Eg|1 to Eg|5, with Eg|5 showing the highest frequency for this space group. These results suggest that the symmetry Cc is more frequent for low band gap values, while the P21/c is more representative in materials with high band gap values. Despite these apparent correlations, no association rules are found between the space groups and Eg, likely due to the reduced number of materials involved in these trends.

Table 3S. Relevant association rules corresponding to distance N-Hal

Rule	Antecedent	Consequent	Conf	Supp	Lift	Odds ratio	Bi-lift
C.1	Nr|1, dNHal|3	Chain|Arom	1	0.11	2.06	1000	2.37
C.2	Eg|5, dNHal|1	Egc|5	0.96	0.12	4.66	202.82	10.18
C.3	dNHal|5	Hal|I	0.94	0.19	1.38	10.22	1.53
C.4	dNHal|4	Hal|I	0.91	0.19	1.34	6.4	1.46
C.5	dNHal|3	Hal|I	0.82	0.16	1.2	2.4	1.26
C.6	dNHal|3	Chain|Arom	0.76	0.15	1.56	4.3	1.8
C.7	dNHal|5	Chain|Ali	0.71	0.15	1.39	2.89	1.54
C.8	dNHal|1	Egc|5	0.67	0.13	3.26	19.28	7.1
C.9	dNHal|1	Eg|5	0.67	0.13	3.26	19.28	7.1
C.10	dNHal|1	Chain|Ali	0.64	0.12	1.24	1.86	1.31
C.11	dNHal|5	Beta|2	0.63	0.13	1.24	1.85	1.32
C.12	dNHal|1	Hal|Br	0.61	0.12	2.47	8.11	3.8
C.13	Beta|2, Chain|Ali, Hal|I	dNHal|5	0.59	0.12	2.87	11.62	5.37
C.14	Hal|I, dNHal|5	Egc|1	0.58	0.11	2.9	11.12	5.29
C.15	dNHal|3	Nr|1	0.58	0.11	1.45	2.46	1.62
C.16	dNHal|2	Chain|Arom	0.57	0.12	1.18	1.55	1.23

The rules presented in Table 3S demonstrate odds ratios greater than 1, indicating a positive association between the antecedent and consequent of these rules. Notably, rules C.1 and C.2 have the highest odds ratios and confidence values, suggesting that all materials classified as dNHal|3 and Nr|1 also contain Chain|Arom. Although rules C.8 and C.7 show low support values, they exhibit high odds ratios, lift, and bi-lift. These rules represent a significant association between Eg|5 and Egc|5 with dNHal|1.

```
#!/usr/bin/env python
# coding: utf-8


import numpy as np
import pandas as pd
import matplotlib.pyplot as plt
import csv
import math
import re
from efficient_apriori import apriori
from sklearn.cluster import KMeans
from sklearn import preprocessing
from sklearn.compose import make_column_selector as selector
from scipy.cluster.hierarchy import dendrogram
from sklearn.cluster import AgglomerativeClustering


#Function to generate the dendrogram
def plot_dendrogram(model, **kwargs):
    # Create linkage matrix and then plot the dendrogram

    # create the counts of samples under each node
    counts = np.zeros(model.children_.shape[0])
    n_samples = len(model.labels_)
    for i, merge in enumerate(model.children_):
        current_count = 0
        for child_idx in merge:
            if child_idx < n_samples:
                current_count += 1  # leaf node
            else:
                current_count += counts[child_idx - n_samples]
        counts[i] = current_count

    linkage_matrix = np.column_stack(
        [model.children_, model.distances_, counts]
    ).astype(float)

    # Plot the corresponding dendrogram
    D=dendrogram(linkage_matrix, **kwargs)
    return(D)
modelo_hclust_ward = AgglomerativeClustering(
                            affinity = 'euclidean',
                            linkage  = 'ward',
                            distance_threshold = 0,
                            n_clusters         =None)
```

```
# Reading the dataset preprocessed

data=pd.read_csv("Aclasesquintdata2perovsprosDistortion_251124corrNHNov2.csv")
data2a=data
#  Prepare Data for apriori algorithm

transactions = data2a.values.tolist()

#Generation of association rules by the apriori algorithm
itemsets, rules = apriori(transactions, min_support=0.1, min_confidence=0.10)

#Creation of the dataframe of association rules
A2a=data2a
A2a["label"]=data["label"]
select="-"
characters =["’","[","]"," ","."]
Selec=[]
rul=[]
conf=[]
supp=[]
lift=[]
ant=[]
conc=[]
for i in rules:
    b=str(i)
    S= str(select) in b
    if S ==True:
        d=b.split("(c")
        N= re.findall(r’-?\d+\.?\d*’, d[1])
        r=d[0].replace(’[’, ’’)
        ant_c=r.split("->")

        rul.append(r)
        conf.append(float(N[0]))

        supp.append(float(N[1]))
        lift.append(float(N[2]))
        ant.append(ant_c[0])
        conc.append(ant_c[1])
        Selec.append(b)

     rules2={
    "regla":rul,
    "antecedente":ant,
    "consecuente":conc,
    "confianza":conf,
    "soporte":supp,
    "ascenso":lift }

   rul=pd.DataFrame(rules2)
```

```
######One-Hot encoding of items to each association rules
 des=[]
 transactions = data2.values.tolist()

 for i in transactions:
     for j in i:
         des.append(j)

 des1=list(set(des))

 # Funtion of generation of column of zeros for item

 def NewC(d,N,dat):
     D=[]
     for i in range(d):
         D.append(0)
     Dat[N]=D

 #Add one when the item is present
 for ia in range(len(des1)):
     NewC(len(rul),des1[ia],rul)
     for ja in range(len(rul)):
         if des1[ia] in str(rul["regla"][ja]):
             rul[des1[ia]][ja]=1

 ldes=list(des1)
 vec=[]
 B=rul[ldes]

 for i in range(len(rul)):
     A=B.iloc[i,:]
     A=np.array(A)
     k=" "
     for j in A:
         a=str(j)
         k=k+a
     vec.append(k)

 rul["vec"]=vec
 rV=rul["vec"].unique()
 idx=[]
 k=0
 # Sorting the rules according to items and lift metric
 for i in rV:
     k=k+1
     #print(k)
     Rm=rul[rul["vec"]==i]
     Rm=Rm.sort_values(by="ascenso",ascending=False)
     idx.append(Rm["Unnamed: 0"].iloc[0])
 rul=rul.iloc[idx]
 rul=rul.reset_index()

 ldes=rul.columns.values
```

```
lo=['Unnamed: 0.1', 'index', 'Unnamed: 0', 'regla', 'antecedente',
     'consecuente', 'confianza', 'soporte', 'ascenso', 'Num_Comp','Comp_lista']
Al=set(ldes)-set(lo)
Al=list(Al)

Al=[]
for i in ldes:
   A=sum(rul[i])
   if A ==0:
      rul=rul.drop(columns=[i])
   if A!=0:
     Al.append(i)

  rul.loc[(rul['simetria|P21']==1) & (rul['simetria|P21/c']==1),'simetria|P21']=0

  # function to list of antecedent and consequent
  def AntConpos(linp,dat,x):
      A1l=[]
      A1=[]
      an=[]
      for ai in range(len(linp)):
         ia=linp[ai].split("|")
         an.append(ia[0])
         if x==1:
            la=dat["Unnamed: 0"][dat[ia[0]]==linp[ai]]
            la=la.reset_index()

        A1.append(la["Unnamed: 0"])


      Hl=A1[0]

      for mi in A1:
         Hl=set(Hl)&set(mi)

      Hl.sort()

     return(Hl)


# Odds ratio and bi-lift calculation

NR=[]
Na=[]
Nc=[]
pAnB=[]
pnAB=[]
pnAnB=[]
Oddr=[]
Bilift=[]
sumaP=[]
pA=[]
pnA=[]
pB=[]
```

```
pnB=[]
for i in range(len(rul)):


    R=[]
    A=[]
    C=[]

    for j in Al:
        if rul[j][i]==1:
              R.append(j)
    for m in R:
        if m in rul["antecedente"][i]:
            A.append(m)

        if m in rul["consecuente"][i]:
            C.append(m)

    A1e=AntConpos(A,data,1)

    cneg=[]
    B1c=AntConpos(C,data,1)


    cneg=set(data["Unnamed: 0"])-set(B1c)
    aneg=set(data["Unnamed: 0"])-set(A1e)



    A1NBi=[]
    for ant in A1e:
        for cb in cneg:
            if ant==cb:
                A1NBi.append(ant)
    A1NBi=list(set(A1NBi))



    NAB1c=[]
    for conc in B1c:
        for ab in aneg:
            if conc==ab:
                NAB1c.append(conc)
    NAB1c=list(set(NAB1c))



    NANB=[]
    for bconc in cneg:
        for abn in aneg:
            if bconc==abn:
                NANB.append(bconc)
        NANB=list(set(NANB))

    pAnB.append(len(A1NBi)/len(data))
```

```
        pnAB.append(len(NAB1c)/len(data))
        pnAnB.append(len(NANB)/len(data))
        pA.append(len(A1e)/len(data))
        pnA.append(len(aneg)/len(data))
        pB.append(len(B1c)/len(data))
        pnB.append(len(cneg)/len(data))
        #Odds ratio metric
        Od=rul["soporte"][i]*(len(NANB)/len(data))/((len(NAB1c)/len(data))*(len(A1NBi)/len(data)))
        #Bilift metric
        Bil=rul["soporte"][i]*(len(aneg)/len(data))/((len(NANB)/len(data))*(len(A1e)/len(data)))
        suma=(rul["soporte"][i])+(len(NANB)/len(data))+(len(NAB1c)/len(data))+(len(A1NBi)/len(data))
        Bilift.append(Bil)
        Oddr.append(Od)
        sumaP.append(suma)
    # Probability of the antecedent
    rul["PA"]=pA
    # Probability that antecedent does not happen

    rul["PnA"]=pnA
    # Probability of the consequent
    rul["PB"]=pB
    # Probability that consequent does not happen
    rul["PnB"]=pnB
    # Probability that the antecedent happens and consequent does not happen

    rul["PANB"]=pAnB

    # Probability that the antecedent does not happen and consequent happens

    rul["PNAB"]=pnAB
    # Probability that the antecedent does not happen and consequent does not happen

    rul["PNANB"]=pnAnB

    #Odds ratio metric
    rul["Oddr"]=Oddr

    #Bi-lift metric
    rul["Bilift"]=Bilift


    item=set(rul.columns.values) - set(other)
    item=list(item)

#Identification of rules by item


 metric2=['confianza', 'soporte', 'ascenso','Oddr', 'Bilift']


    Lat=["Ginterc|0","Alpha|2","Cinter|0","Phase|else","S2D|100","Nlay|1","Ang90|4",'NCr|0','Nr|0',"NN|1",
    "M|P b","Qc|1","Hal|I"]

    for i in Lat:
        rul=rul[rul[i]!=1]
```

```
  rulm=rul
  #Items to identify in the association rules set
  t=['Eg|5','Eg|1','Eg|2','Eg|3','Eg|4']

  rul2=rulm[(rulm[t[0]]==1)|(rulm[t[1]]==1)|(rulm[t[2]]==1)| (rulm[t[3]]==1) |(rulm[t[4]]==1)]


#Selection relevant rules
#Standardization of metrics

  mm_scaler=preprocessing.MinMaxScaler()
  Namerulm=list(set(rul2["regla1"]))
  data_s1=mm_scaler.fit_transform(rul2[metric2])
  data_s1= pd.DataFrame(data_s1,columns=metric2)

  Data_cat1a=data_s1
  namerow1=Namerulm

#Building of dendogram

  modelo_hclust_ward.fit(X=Data_cat1a)
  model1a = modelo_hclust_ward.fit(Data_cat1a)
  fig = plt.subplots(1, 1, figsize=(8, 6),layout="constrained")
  plt.title("Eg dendrogram ",fontsize=10)
  plot_dendrogram(model1a, labels=namerow1)
  plt.ylabel("Cluster distance",fontsize=10,rotation='vertical')

  D=plot_dendrogram(model1a, labels=namerow1)
  plt.xticks(fontsize=10,rotation='vertical')
  plt.yticks(fontsize=10)

  #Identify of clusters of dendogram
  DL=list(D.values())
  LabC=D['leaves_color_list']
  idx=D['leaves']
  LabC1=[]
  for jm in range(len(rul2["regla1"])):

     for im in range(len(LabC)):


           if idx[im]==jm:
            LabC1.append(LabC[im])

  rul2["No Cluster"]=LabC1
  plt.savefig("Egalldendometric2nov2.jpg", dpi=300)
  rul2.to_csv("Egallrulesfilmod.csv")
```
